# A qualitative study to explore perceptions of exercise as a smoking cessation tool among people with HIV who smoke cigarettes

**DOI:** 10.18332/tpc/221807

**Published:** 2026-07-10

**Authors:** Nicholas J. SantaBarbara, Joshua St. Louis, Warren Scott Comulada, Jennifer Cantrell, Hope A. Finlayson, Alysse G. Wurcel, Kristine M. Erlandson

**Affiliations:** 1 Department of Athletic Training and Exercise Science, School of Nursing and Health Science, Merrimack College, North Andover, United States; 2 Department of Family Medicine, Tufts University School of Medicine, Boston, United States; 3 Department of Psychiatry and Biobehavioral Sciences, Fielding School of Public Health, Los Angeles, United States; 4 Department of Health Policy and Management, Geffen School of Medicine, University of California, Los Angeles, United States; 5 Department of Social and Behavioral Sciences, School of Global Public Health, New York University, New York, United States; 6 Clinical Mental Health Counseling, Merrimack College North Andover, North Andover, United States; 7 Department of Infectious Disease, Adult Primary Care, Boston Medical Center, Boston, United States; 8 Department of Medicine-Infectious Disease, University of Colorado Anschutz, Aurora, United States

**Keywords:** HIV, AIDS, cigarette smoking, physical activity, exercise

## Abstract

**Introduction:**

Cigarette smoking remains disproportionately prevalent among people with HIV (PWH) and contributes substantially to preventable morbidity and mortality. Although exercise confers many health benefits, little is known about how PWH who smoke perceive exercise as a strategy to support smoking cessation. The purpose of this qualitative study was to explore barriers and facilitators to smoking cessation and exercise engagement, with particular attention to exercise as a potential cessation-support strategy.

**Methods:**

Within this qualitative study, PWH were recruited from HIV clinics in Colorado and New England between September 2025 and February 2026, and were eligible if they were aged ≥21 years and current smokers. Guided by the Health Belief Model (HBM) and the Social Ecological Model (SEM), semi-structured interviews were transcribed verbatim and analyzed using a hybrid deductive–inductive framework.

**Results:**

Ten PWH (50.6 ± 8.8 years) participated. Participants demonstrated high awareness of smoking-related harms yet described persistent individual and structural barriers to quitting. Exercise was frequently perceived as a supportive strategy that reduced cravings, improved mood, and provided behavioral structure, particularly when embedded in socially supportive contexts. Multilevel barriers included addiction, stress, physical limitations, limited organizational attention to exercise, and resource constraints. Facilitators included peer accountability, structured group exercise, supportive counseling, and environmental incentives. Emergent themes highlighted behavioral substitution and autonomy as important.

**Conclusions:**

PWH who smoke perceive exercise not only as a health behavior but as a potential cessation-support strategy when delivered within socially connected and autonomy-respecting environments. Additional work is needed to develop multidisciplinary interventions that may enhance smoking cessation efforts.

## Introduction

Cigarette smoking remains the leading cause of premature and preventable mortality in the US^1^ and is highly prevalent among people with HIV (PWH)^2^. Approximately 40–60% of PWH in the US currently smoke cigarettes, a rate two to three times higher than that observed among people without HIV (PWoH)^3^. The health consequences of smoking are also disproportionately severe for PWH. Compared with PWoH who smoke, PWH who smoke experience higher rates of cardiovascular and pulmonary disease and face a substantially increased risk of certain cancers, including lung and tracheal cancer^4-6^. Although more than two-thirds of PWH express interest in quitting smoking^7^, cessation success is lower and relapse rates are higher compared with PWoH^3^, reflecting the complex and multidimensional challenges this population faces^8,9^.

Understanding barriers and facilitators to smoking cessation among PWH is essential for developing effective interventions that improve both HIV-related and overall health outcomes while reducing preventable morbidity and mortality. Prior research has identified numerous individual and contextual factors that contribute to continued smoking among PWH. For example, qualitative work among HIV service providers has highlighted gaps in patient awareness regarding the compounded health risks associated with smoking and HIV^9^. Other studies among PWH who smoke have documented a high prevalence of co-occurring conditions, including substance use disorders and depression^8-10^. Additional barriers include high nicotine dependence, use of smoking as a stress management strategy, low self-efficacy for quitting, and social environments in which smoking is normalized^11^.

Despite these challenges, smoking cessation remains one of the most impactful actions PWH can take to improve long-term health outcomes. Some researchers have argued that quitting smoking may rival the health benefits of antiretroviral therapy adherence^12^. PWH who quit smoking experience lower mortality rates compared with those who continue to smoke, with one study reporting life expectancy gains of up to 5.7 years and reduced incidence of AIDS-related events, cardiovascular disease, and non-AIDS-related cancers^13^. However, long-term cessation success remains low, as a recent Cochrane review reported sustained abstinence rates below 15% among PWH receiving behavioral support, pharmacotherapy, or a combination of the two^14^. In addition, uptake of these evidence-based treatments remains low, as many PWH attempt to quit smoking without medical assistance^15^.

The U.S. Department of Health and Human Services recommends exercise as an adjunct behavioral approach for individuals attempting to quit smoking^16^. Exercise offers numerous physical and mental health benefits for PWH and does not adversely affect HIV disease progression^17,18^. Exercise can reduce smoking behavior through several psychological and physiological mechanisms, including reducing nicotine cravings and withdrawal symptoms, improving mood, alleviating psychological distress, and supporting weight management^19-21^. Although this experimental evidence is found primarily in PWoH, one cross-sectional study found that PWH who are more physically active have lower odds of smoking^22^.

Despite these potential benefits, little is known about whether PWH who smoke perceive exercise as a viable or relevant strategy to support smoking cessation. Most existing research has focused on the physiological benefits of exercise or on associations between exercise and smoking behavior, rather than on the lived experiences and perspectives of PWH themselves^22,23^. Given the unique psychosocial, structural, and health-related challenges faced by PWH, it cannot be assumed that exercise is viewed as feasible, acceptable, or meaningful within the context of smoking cessation. Understanding how PWH who smoke conceptualize exercise, whether as a supportive strategy, an unrealistic recommendation, or something else entirely, is critical for designing interventions that are both effective and responsive to patient needs and preferences.

## Methods

### Study design

This qualitative study was conducted with adult PWH between September 2025 and February 2026. Guided by the Social Ecological Model (SEM) and the Health Belief Model (HBM), this qualitative study explored how PWH who currently smoke perceive smoking cessation and exercise, with particular attention to exercise as a potential tool to support quitting. Specifically, we aimed to identify multilevel barriers and facilitators influencing both smoking cessation and exercise engagement and to understand whether and how participants conceptualize exercise as a viable cessation strategy. Study procedures were approved by the Institutional Review Board (IRB) of Merrimack College.

### Participants

Adult PWH were recruited from HIV clinics in Colorado and Massachusetts. Individuals were eligible if they were aged >21 years, were currently smoking cigarettes, living with HIV, and had access to a phone or computer for the interview.

### Procedures

Participants were recruited via word of mouth (i.e. from co-authors) and flyers posted in clinics in (Colorado and Massachusetts). Participants self-reported eligibility by scanning a QR code embedded in study flyers, which linked to an online Research Electronic Data Capture (REDCap) questionnaire. Participants reviewed the study information and provided virtual consent through REDCap prior to being scheduled for their interview. Participants completed a brief demographic survey to report age, gender, race/ethnicity, sexual orientation, education level, employment, and income. Interviews were conducted via telephone or videoconferencing with optional video (e.g. Zoom), per participant preference, and participants had the choice to be interviewed in Spanish or English. Before the interview, participants were given the opportunity to ask questions about the study. Upon completion of the interview, participants received a $20 electronic gift card via email or text message.

### Semi-structured interview

The interview questions were guided by both the HBM and SEM^24,25^. The HBM posits that motivation to promote health and prevent disease is influenced by several key factors: perceived susceptibility, severity, benefits, barriers, and cues to action^24^. The SEM seeks to understand the individual, interpersonal, organizational, community, and public policy factors that influence health and health behaviors^25^. The full interview guide can be found in Table 1.

**Table 1:** Example interview questions using the Health Belief Model and the Social Ecological Model for a remote qualitative study that explored perceptions, barriers, and facilitators, of smoking cessation and exercise among ten PWH who smoke

Models	Questions
**Health Belief Model**	
**Perceived susceptibility**	In general, what do you believe are the biggest health concerns you face as a person with HIV who is currently smoking?
**Perceived severity**	How severe do you believe the health consequences are of smoking?
**Health motivation**	How do you think exercise could help you quit smoking?
**Perceived benefits**	How do you think quitting smoking and exercising could reduce your risk of getting sick?
**Perceived barriers**	Who or what has prevented you from quitting smoking and starting to exercise?
**Cues to action**	Who or what has impacted you the most when it comes to wanting to quit smoking and start exercising?
**Self-efficacy**	How confident are you at starting and maintaining an exercise routine?
**Social Economical Model**	
**Individual level**	What barriers do you face to quitting smoking and exercising?
**Interpersonal level**	What role do your family and friends play in helping you quit smoking and exercising?
**Organizational level**	What does your organization do to help you quit smoking and exercising?
**Community level**	How could your community better support your efforts to quit smoking and start exercising?
**Policy level**	What laws or policies could be put into place to make it easier to quit smoking and exercise?

PWH: people with HIV.

### Data analysis

Demographic and descriptive characteristics are presented as frequencies and percentages for categorical outcomes and means, standard deviations, and ranges for continuous outcomes. Data collection proceeded concurrently with analysis, and recruitment continued until thematic redundancy was observed. After the eighth interview, no new codes were identified, and subsequent interviews (i.e. 9 and 10) confirmed code saturation. This aligns with empirical evidence suggesting that code saturation in focused qualitative studies with relatively homogenous samples often occurs within 9 to 12 interviews^26^.

Interviews were audio-recorded, transcribed verbatim, and entered into QSR NVivo (version 14) for analysis. Interviews were conducted by two team members trained in qualitative interviewing methods. Interviewers had no prior relationship with participants. All interviewers completed mock interviews to promote consistency in data collection. A certified medical translator conducted interviews in Spanish when preferred by participants and translated the resulting data into English. The translator received training in study procedures, including multiple mock interviews with the first "author, Nicholas J. SantaBarbara," to promote consistency and enhance the trustworthiness of the data. We used the framework method to conduct a hybrid deductive–inductive thematic analysis^27^. An initial codebook was developed deductively based on the interview guide and key constructs from the HBM and SEM. Two members of the research team (NJS and HAF) independently reviewed transcripts to familiarize themselves with the data and apply the preliminary coding structure. As coding progressed, additional codes were added inductively to capture themes emerging from participant narratives that were not fully represented within the initial HBM and SEM frameworks. We reviewed each theme to make sure the ideas within it were consistent and distinct from other themes. Discrepancies were resolved through discussion until consensus was reached. Themes were considered present when participants explicitly described experiences consistent with a given construct, and exemplar quotations are provided to reinforce these themes.

## Results

Thirty-six adults with HIV initially expressed interest in the study. Of these individuals, 20 self-reported that they were not current cigarette smokers, four could not be reached to schedule their interview, one self-reported not having an HIV diagnosis, and one declined further participation. The 10 PWH who completed the interview had a mean age of 50.6 (8.8) years, 7 males, 6 White, Non-Latino/a and 4 Latino(a). Table 2 summarizes the sample demographics and smoking and exercise behaviors.

**Table 2:** Characteristics of participants who completed a remote qualitative study that explored perceptions, barriers, and facilitators, of smoking cessation and exercise among PWH who smoke (N=10)

Characteristics	n (%)
**Age** (years), mean (SD), range	50.6 (8.8), 37–60
**Gender**	
Male	7 (70)
Female	3 (30)
**Race**	
Black/African-American	2 (20)
White/Caucasian	6 (80)
**Ethnicity**	
Hispanic/Latino	4 (40)
Non-Hispanic/Latino	6 (60)
**Sexual orientation**	
Gay	3 (30)
Straight	5 (50)
Bisexual	1 (10)
Don’t want to answer	1 (10)
**Education level**	
Lower than high school	3 (30)
High school graduate or higher	7 (70)
**Employment**	
Full-time	3 (30)
Other	7 (70)
**Income ($)**	
<25000	4 (40)
≥25000	4 (40)
Do not know	2 (20)
**Smoking behaviors**	
**Average cigarettes smoked per day**, mean (SD), range	11.1 (6.2), 5–20
**Average years as a smoker**, mean (SD), range	31.7 (10.5), 14–43
**Current exercise behaviors**	
**Frequency** (days/week)	
0	2 (20)
2–3	5 (50)
5–6	1 (20)
7	1 (10)
**Type**	
Aerobic	6 (60)
Resistance	1 (10)
Other	1 (10)
**Duration** (minutes/exercise bout)	
<30	4 (40)
≥30	4 (40)

Several themes emerged from the interviews, including key facilitators that may support reductions in smoking behavior and increases in physical activity, as summarized below.

### Perceptions of smoking risk and severity

Participants widely recognized the health risks associated with smoking, particularly cancer and respiratory disease, while others described broader health effects:

*‘Cancer. You have to be more careful with cancer because our body system is not as before [being diagnosed with HIV]. It will be a little bit harder for us to fight’* (Participant 2)

*‘It affects everything* … *It especially affects exercises because I am a person who likes to exercise, but I can't do it like before because I am smoking. My personal life is affected too, cigarettes are not pleasant at all, I would say. It doesn't help your health. Let’s say that you live less because of smoking, because it gives you cancer. Many worries because I am thinking about what is going to happen to me, it is difficult, it is not the same as saying that one is going to stop because it is different.’* (Participant 3)

*‘*… *and in the womb they gave me ablation to cauterize the cancer cells in the cervix. I was told that this is caused by cigarettes, and if I continue, I will die.’* (Participant 6)

Despite acknowledging severity, participants described difficulty translating awareness into sustained cessation:

*‘Yes, of course! I want to make an effort [to quit smoking]* … *but my body asks me for cigarettes.’* (Participant 1)

### Perceived benefits of quitting and the role of exercise

Participants articulated clear perceived benefits of smoking cessation, including improved breathing, increased energy, reduced disease risk, financial savings, and improved ability to exercise:

*‘It would be good for my health because I will be able to exercise more. I would be healthier, I would eat a little bit more, you know, and I would do other things good for my health that I don't do now, like exercise. Sometimes I'm hungry, but instead of eating something, I smoke a cigarette, and my hunger goes away. I would say that I lose my appetite, and things like that. I would say that cigarettes keep me awake, too, and things like that. And that is not good at all for me.’* (Participant 3)

Exercise was viewed as beneficial not only for physical health but also for mental well-being, stress reduction, social connection, and as a distraction from cravings. Many participants reported smoking less or not at all during periods of physical activity, reinforcing the perceived value of exercise as a supportive cessation strategy:

*‘When I exercise, I do not smoke. When I am exercising, I feel like I can quit, I don't smoke at that time.’* (Participant 1)

*‘I was thinking to do more of that [walking] with my son and his baby. And it helps me not think, and we enjoy them with my dog. And when I'm walking with my dog and my granddaughter, I don't even remember the cigarette.’* (Participant 6)

However, for others, exercise and smoking coexisted:

*‘I do think it [exercise] helps, but I don’t mind the fitness because I think is the same problem, when I get home from the gym, you know, I have a cigarette, it is ridiculous, but it is almost like if I ... I don't know, my payoff for going to the gym I think is to have a cigarette.’* (Participant 5)

### Cues to action

Cues to action included health crises (e.g. cancer diagnoses, hospitalization), financial strain, pregnancy, and accountability to family members:

*‘I can't afford them cigarettes, they’re expensive.’* (Participant 7)

*‘When I realized I was pregnant I said to myself: “I have a human being inside me”* … *I stopped until after I gave birth’*. (Participant 2)

### Multilevel barriers

#### Individual-level barriers

Participants identified nicotine addiction, stress, anxiety, depression, fatigue, physical limitations, and low motivation as primary barriers to quitting smoking and engaging in exercise. Several participants described smoking as a coping mechanism for loneliness and trauma:

*‘I would say life stress, work, all of that* … *a habit is really hard to break.’* (Participant 5)

*‘What prevents me from quitting smoking* … *anxiety* … *when I get anxious the first*

*thing I do is lighting up the cigarette.’* (Participant 6)

Exercise barriers included shortness of breath, asthma, fear of exacerbating symptoms, and lack of confidence and knowledge (i.e. on how to and how much exercise to do) following illness or deconditioning:

*‘... I like it [exercise], it’s good, it is a nice stuff, but I start coughing when I do it. Once I start exercising, I stop because of the phlegm. I start coughing, I start feeling my lungs breaking apart.’* (Participant 3)

*‘My health is poor* … *Also, I was feeling weak, I was having lung issues and my back hurting as well. I was having crampy pain on my legs, and my calcium level was very low. I was feeling numbness on my legs.’* (Participant 6)

#### Interpersonal-level barriers and supports

Participants reported limited interpersonal support for smoking cessation, including social networks that normalized smoking. Group-based exercise and peer accountability were repeatedly described as more motivating than exercising alone:

*‘Well, you know, when I was trying to quit smoking, it was just more stress and environment. Yeah. You know, it was just I was in an investment environment where just people smoked and it was at work and it was just a thing you did. And I think it was also stress. And it was also, I wouldn't say a barrier, but it was, you know, it was just social. Plus, I had a boyfriend that was smoking. Right. So it was all around me.’* (Participant 8)

Conversely, interpersonal support particularly from children, partners, peers, or religious communities, emerged as a strong facilitator when present:

*‘I think it was the time when I was in college and I have friends who didn't smoke at that time, so I think when I was hanging out with them I didn't want to be the only one smoking around because as I said, not everyone likes cigarette smell or someone smoking next to you. And I decided to stop for a while. My mom also smokes and at that time I was living alone just with my cousins, and they smoked too but we decided to quit together. The fact that being with them motivated me to stop. Having a person nearby with the same situation who wants to quit smoking motivates you and I wanted to quit smoking. This helped me quit smoking back then.’* (Participant 3)

#### Organizational-level influences

Participants reported that healthcare organizations emphasized medication management while providing minimal guidance on exercise. Smoking cessation support was often limited to nicotine replacement therapy (NRT; patches) or brief advice, which participants perceived as insufficient. Participants explicitly expressed frustration at the absence of group-based cessation programs or peer-led support within clinical or community organizations:

*‘Never not once* … *has exercise ever been. something that they bring up. In fact, you know, they talk about mental health, but nobody ever talks about physical health.’* (Participant 9)

*‘It seems like every time I go [to the doctors], they focus on blood tests and diabetes and pre-diabetes and cholesterol. And then you take a medicine. But now that were talking, there’s never been a situation where they've discussed exercise and whatever exercise looks like.’* (Participant 8)

#### Community-level barriers

Community-level barriers included lack of accessible exercise facilities, limited recreational programming, financial constraints, housing, and discomfort using gyms due to stigma or lack of instruction. However, some described these resources as underutilized or inaccessible due to cost:

*‘Yeah and as I walk around, you know, the nearby parks are like very small. There’s a dog park, there’s another park, you know, it’s about, I don't know, a tenth of a block is the biggest park around here. And there’s really nothing to do there except for people watch.’* (Participant 9)

#### Policy-level influences

Beyond individual and interpersonal influences, participants emphasized the role of policy-level conditions in shaping smoking behavior. Smoke-free regulations in public spaces and housing environments were perceived as reducing opportunities to smoke, while increased cigarette prices functioned as a financial pressure to quit:

*‘My environment supports non-smoking because it’s very, it’s just recognized not only not that good for health, but also, there’s shame involved with it [smoking].’* (Participant 8)

*‘No smoking areas around hospitals, clinics, bus stops—those help. Same with businesses that ban smoking.’* (Participant 7)

*‘Make it $20 bucks a pack* … *and give that money to cancer research. Just limit the opportunity for someone to want to pick up smoking.’* (Participant 8)

### Facilitators of smoking cessation and exercise engagement

Across ecological levels, facilitators included peer support from individuals with lived experience, structured group-based exercise, non-judgmental counseling, and tangible incentives such as grocery cards or subsidized gym access. Participants consistently emphasized that supportive, stigma-reducing environments were more effective than punitive or purely didactic approaches:

*‘Having someone to walk with me would be a huge help. A set time 3 or 4 or 5 times a week. If I have a personal commitment, I will do it. If left to myself, I probably won't. I still like the idea of a Smokers Anonymous group as well.’* (Participant 10)

*‘I think they could have group sessions. I think they should create group sessions like alcoholic would. Just like drug addict would, or pill person would. Why don’t they have group sessions for people who smoke cigarettes? There is none out there.’* (Participant 2)

*‘I will give incentives for people who can be without smoking for certain period. Incentives like a free gym membership for a year, you know what I mean*?*’* (Participant 4)

### Emergent themes beyond HBM and SEM

In addition to themes aligned with the HBM and SEM, several inductively derived themes emerged that were not fully captured by these frameworks, such as autonomy dynamics, coping substitution, and perceived gaps in healthcare delivery.

#### Autonomy and reactance

Participants emphasized the importance of autonomy in behavior change and that supportive, nonjudgmental approaches may be more effective than directive messaging:

*‘If you say, you must do this, I can find a hundred ways to say, oh, no, I will not.’* (Participant 4)

*‘I'm more comfortable having suggestions and getting information, opportunities. If somebody says, you have to exercise, I'm going to go have a cigarette.*’ (Participant 9)

#### Behavioral substitution and emotional coping

Smoking was frequently described as a coping mechanism for stress, anxiety, and loneliness. Others described replacing smoking with food, gum, or exercise. These suggest that cessation requires not only eliminating nicotine use, but replacing the regulatory function smoking serves:

*‘What prevents me from quitting smoking, as I told you, the problems sometimes and anxiety prevent me because when I get anxious the first thing I do is lighting up the cigarette. But, as I told you, I am thinking to quit and I'm ready and put some willpower and go to church, attend church programs. I also have a little plant, I take care of it, I water it, I talk to it and I feel like that helps me and keeps me distracted. I am trying to live with positive things in my life and try to forget the cigarette.’* (Participant 6)

*‘I did transfer my smoking to eating. That’s what I do to kind of compensate with anxiety or just, you know, I'm tired, I'm exhausted, I'm frustrated, I'm older, I'm single. And so food, you know, it just replaced cigarettes.’* (Participant 8)

#### Perceived institutional gaps

Participants consistently reported frustration with a lack of guidance on how to quit or exercise, that exercise was rarely integrated into HIV care discussions:

*‘They [doctors], you know, offer me every opportunity, and I know they will do whatever they can do to get me to quit ... I just don't know how to. I've tried it all. So, they're offering the Chantix, the lozenge, the patches, the hypnotherapy* … *they bring it [exercise] up and they encourage me to do more. and, you know, they're very supportive and trying to help me find ways to quit. I'm just at a loss that I don't know how. And with exercise, the same thing.’* (Participant 10)

*‘I have never seen people on the streets giving away patches or flyers to quit smoking. I have not seen that here. I have not seen any of that, no programs, nothing. I tell you the truth, nothing at all.’* (Participant 1)

## Discussion

This qualitative study examined how PWH who smoke conceptualize exercise as a potential smoking cessation strategy. While prior research has documented high smoking prevalence, multilevel barriers to cessation, and low long-term abstinence rates among PWH^2,3^, little is known about whether exercise is perceived by PWH as a meaningful cessation tool. By integrating the HBM and SEM, this study extends existing literature by highlighting exercise not simply as a health behavior, but as a participant-endorsed cessation support strategy.

Participants frequently described smoking less or not at all during exercise, suggesting that exercise functioned as a potential substitute for the reinforcing effects of smoking. Rather than emphasizing only long-term health benefits, participants highlighted immediate improvements in mood, breathing, and urge suppression, aligning with prior evidence that exercise can reduce withdrawal symptoms and improve affect regulation during cessation^21^, while adding a patient-centered perspective demonstrating that PWH themselves recognize this mechanism.

Across interviews, participants demonstrated strong awareness of smoking-related harms, particularly cancer, cardiovascular disease, and respiratory complications including difficulty exercising due to respiratory symptoms. However, consistent with prior cessation literature, risk awareness alone did not translate into sustained quitting^15^, as participants remained current smokers despite recognizing the dangers of smoking. Participants described nicotine dependence, emotional distress, and social stressors that undermined quit attempts. Within the HBM framework, these findings suggest that although perceived susceptibility and severity were high, persistent barriers like addiction and stress-related coping continued to drive smoking behavior. While lifelong abstinence remains the goal, reducing smoking and its co-occurring health risks should not be overlooked^28^. In this context, exercise may represent a complementary harm-reduction strategy for individuals who are unable to achieve complete cessation.

Consistent with the SEM, barriers occurred across individual, interpersonal, organizational, and policy levels. Individual factors included addiction, stress, depression, and physical limitations. Interpersonally, social networks sometimes normalize smoking but could also facilitate behavior change when supportive. At the organizational level, participants reported that HIV care prioritized medication management over lifestyle behaviors such as exercise. While NRT was often available, guidance on its use and counseling about physical activity were rarely discussed during routine care. Prior research suggests many healthcare providers inconsistently deliver cessation counseling due to time constraints, competing clinical demands, and limited training in tobacco treatment^9,29^.

Peer-based and group-oriented supports emerged as the most consistently endorsed facilitators of both smoking cessation and exercise engagement. Participants emphasized the value of interacting with others who had lived experience with smoking, HIV, and behavior change, often expressing skepticism toward purely clinician-driven approaches. Group exercise, shared accountability, and non-judgmental encouragement were described as particularly motivating, consistent with evidence that social support improves adherence to smoking cessation and exercise interventions among PWH^30^. Structural supports such as smoke-free housing, reduced access to cigarettes, and contingency management also served as external cues that reinforced behavior change.

### Strengths and limitations

This study has several strengths, including theory-informed analysis and the novelty of exploring perspectives on exercise for smoking cessation among PWH who currently smoke. However, limitations should be noted. First, the qualitative study design is unable to establish causal relationships between exercise and smoking cessation. Second, the purposive sampling limits generalizability, and participants were recruited from specific geographical and clinical contexts that may influence access to resources and support, which may have resulted in potential selection bias. Nonetheless, the consistency of themes across participants strengthens confidence in the findings.

## Conclusions

This study identified key factors influencing smoking behaviors among PWH, including the role of social support, preferences for autonomy in behavior change, and the influence of structural barriers such as transportation, cost, and environmental conditions. Participants emphasized the importance of supportive, nonjudgmental approaches that align with their readiness for change. Future research is needed to further examine how these factors can be addressed in efforts to support smoking reduction and cessation. Approaches such as motivational interviewing, peer support, and technology-delivered modalities may represent promising areas for investigation, particularly in their potential to address both individual preferences and structural barriers^30,31^. Additional studies, including longitudinal and intervention-based research, are necessary to determine the effectiveness of these approaches in promoting sustained behavior change among PWH.
